# Small RNAs, emerging regulators critical for the development of horticultural traits

**DOI:** 10.1038/s41438-018-0072-8

**Published:** 2018-09-17

**Authors:** Chengjie Chen, Zaohai Zeng, Zongrang Liu, Rui Xia

**Affiliations:** 1 State Key Laboratory for Conservation and Utilization of Subtropical Agro-Bioresources, South China Agricultural University, Guangzhou, 510642 China; 2 Key Laboratory of Biology and Germplasm Enhancement of Horticultural Crops in South China, Ministry of Agriculture, South China Agricultural University, Guangzhou, 510642 China; 3 Guangdong Litchi Engineering Research Center, College of Horticulture, South China Agricultural University, Guangzhou, 510642 China; 4 Appalachian Fruit Research Station, Agricultural Research Service, United States Department of Agriculture, Kearneysville, WV 25430 USA

## Abstract

Small RNAs (sRNAs) have been recently recognized as key genetic and epigenetic regulators in various organisms, ranging from the modification of DNA and histone methylations to the modulation of the abundance of coding or non-coding RNAs. In plants, major regulatory sRNAs are classified as respective microRNA (miRNA) and small interfering RNA (siRNA) species, with the former primarily engaging in posttranscriptional regulation while the latter in transcriptional one. Many of these characterized sRNAs are involved in regulation of diverse biological programs, processes, and pathways in response to developmental cues, environmental signals/stresses, pathogen infection, and pest attacks. Recently, sRNAs-mediated regulations have also been extensively investigated in horticultural plants, with many novel mechanisms unveiled, which display far more mechanistic complexity and unique regulatory features compared to those studied in model species. Here, we review the recent progress of sRNA research in horticultural plants, with emphasis on mechanistic aspects as well as their relevance to trait regulation. Given that major and pioneered sRNA research has been carried out in the model and other plants, we also discuss ongoing sRNA research on these plants. Because miRNAs and phased siRNAs (phasiRNAs) are the most studied sRNA regulators, this review focuses on their biogenesis, conservation, function, and targeted genes and traits as well as the mechanistic relation between them, aiming at providing readers comprehensive information instrumental for future sRNA research in horticulture crops.

## Genetics: Tiny keys to important traits

Studying small RNAs in horticultural plants may reveal the genetic mechanisms underlying many unique and valuable traits, and help improve plant breeding programs. Small RNAs, short RNA molecules only 20–24 bases long, switch genes on or off to control many aspects of plant growth, development, and reproduction. They are well-studied in model plants, but are only just being investigated in horticultural species. In a review of small RNA research in horticultural plants, Rui Xia at South China Agricultural University and co-workers report that horticultural plants contain many novel sRNAs that hold the keys to important traits such as juvenile-to-adult transition in fruit trees, fruit size, fruit quality, and disease resistance. Better understanding of these miRNAs may help in engineering varieties with earlier production of tastier fruit, as well as enhanced resistance to disease.

Since the first plant small RNA (sRNA) was excavated in Arabidopsis in 2002^1^, numerous sRNAs have been found to orchestrate diverse biological processes critical for plant growth, development, and stress responses. Plant sRNAs are a class of short regulatory RNAs of 20–24 nucleotides in length^2,3^. According to their biogenesis and function mechanism, sRNAs are classified into two general types, microRNAs (miRNAs) and short interfering RNAs (siRNAs)^4,5^. Although much of our knowledge regarding sRNAs comes from model plants like Arabidopsis, ongoing research progressively extends into non-model systems, including a group of economically important plants—horticultural plants. These studies greatly expand our understanding of biogenesis, metabolism, and function of sRNA in crops. Here we aim to summarize these sRNA research progress to provide an overview of sRNA-involved regulatory networks vital for the development of critical economic traits in horticultural plants. We focus our review on miRNA and phasiRNA (a class of siRNAs), as they are the most widely studied classes of sRNAs in the recent decades.

## Overview of sRNA research in plants

### Biogenesis of miRNA and phasiRNA

**Fig. 1:**
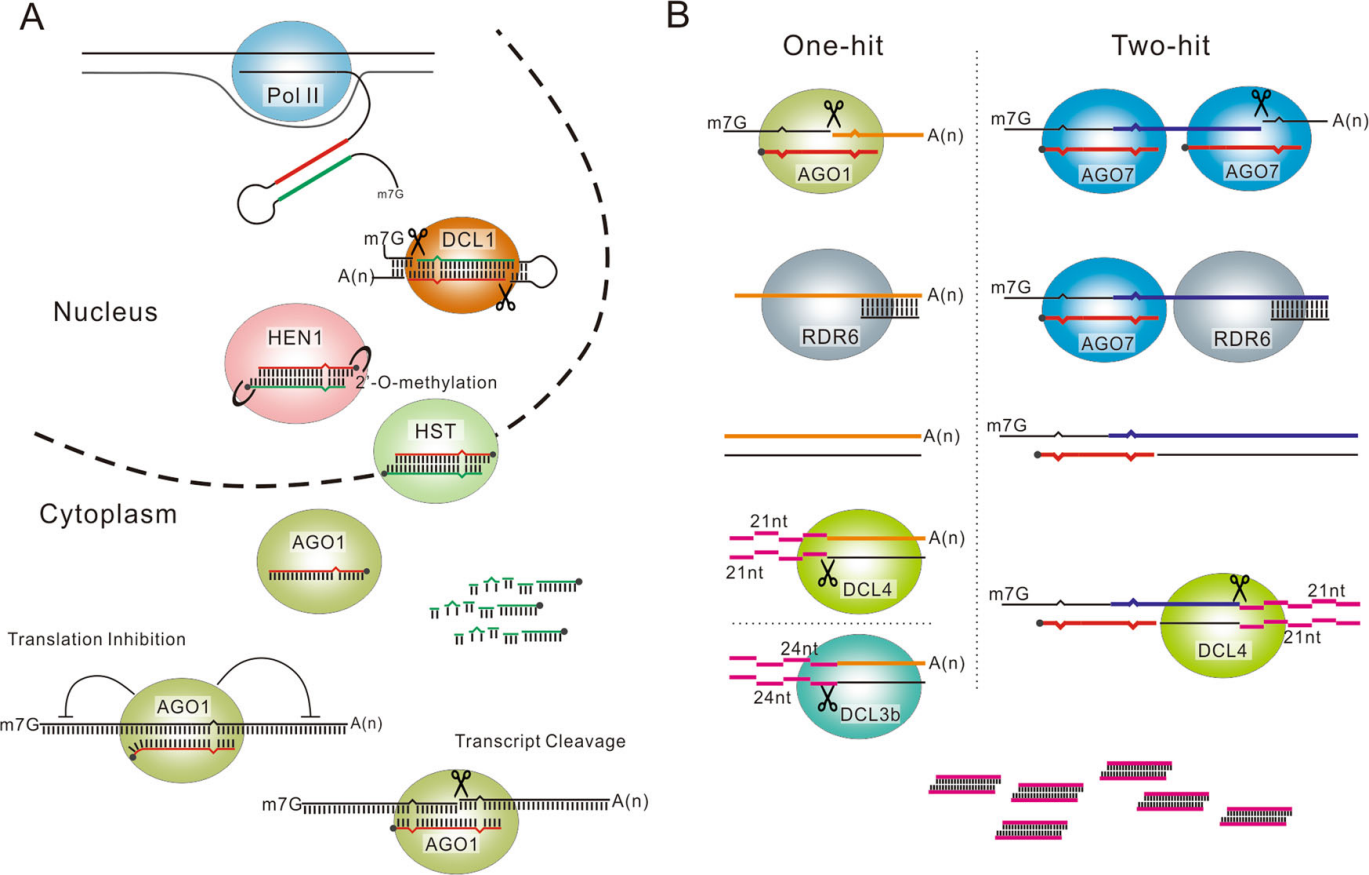
Biogenesis of miRNA and phasiRNA. **a** General process of miRNA biogenesis. A primary miRNA is transcribed from a *MIRNA* locus by pol II, generating a precursor *MIRNA* with a hairpin structure. DCL1 processes the hairpin into a duplex of miRNA (red) and miRNA* (green) with 2-nt 3′-terminal overhangs. HEN1 methylates the 3′-terminal ribonucleotide of the duplex. Subsequently, HST transports the duplex from nucleus to cytoplasm. miRNA* is rapidly degraded while miRNA is loaded into AGO1 in a RISC complex to perform its function, via translation inhibition or transcript cleavage. **b** Two typical modes of phasiRNA biogenesis. In one-hit mode, dsRNA synthesized by RDR6, after the miRNA-mediated cleavage on the single site of target transcript, are processed into 21- or 24-nt phasiRNAs by DCL4 or DCL5 from 5′ to 3′. In two-hit mode, a transcript containing two miRNA target sites is cleaved by miRNA-AGO7 complex at the 3′-terminal target site. The upstream fragment is converted into dsRNA by RDR6 for the generation of phasiRNAs through sequential cleavages by DCL4 from 3′ to 5′

miRNAs are the most functionally important and most studied class of sRNAs in plants, and their biogenesis is an intricate process, widely conserved in plants (Fig. 1a). In brief, a miRNA gene (*MIR*) is firstly transcribed by RNA POLYMERASE II (Pol II) and produces a 5′ capped and 3′ polyadenylated primary transcript (pri-miRNA) with a self-complementary foldback structure (Fig. 1a)^2,6–8^. The pri-miRNA is sequentially sliced by the RNase III endoribonuclease DICER-LIKE1 (DCL1) to yield a miRNA/miRNA* duplex with two-nucleotide 3′ overhangs^2,6–8^. The duplex is then 2-O-methylated at the 3′ terminal residues by HUA ENHANCER1 (HEN1)^9^ and transported from nucleus to cytoplasm by HASTY(HST)^2,6–8^. In the cytoplasm, the duplex is separated with the miRNA* rapidly degraded and the mature miRNA incorporated into the ARGONAUTE 1 (AGO1) protein to form an active RNA-INDUCED SILENCING COMPLEX (RISC)^2,6–8^. After that, the miRNA, requiring almost perfect sequence complementary, guides the RISC complex to regulate its target genes through either transcript cleavage or translation inhibition (Fig. 1a)^2,6–8^.

Phased siRNAs (phasiRNAs) are a special class of siRNAs, which is only found in plants, to date. Its biogenesis relies on the cleavage mediated by sRNAs (mostly miRNA). There are two modes of phasiRNA biogenesis reported so far according to the number of sRNA target sites on the target gene, one-hit and two-hit. In the one-hit mode, a 22-nucleotide (nt) miRNA cleaves its target transcript into two fragments on a single site. The cleaved fragment downstream to the target site is converted into a double-strand RNA (dsRNA) by RNA-DEPENDENT RNA POLYMERASE 6 (RDR6)^6^, and then the dsRNA was chopped by a Dicer protein (DCL4 or DCL5 in grasses) from 5′ to 3′ in a continuous head-to-tail manner, producing dozens of phasiRNAs of certain length (21-nt for DCL4 and 24-nt for DCL5)^10^. In the two-hit mode, a target transcript possesses two target sites, as typified in *TAS3* genes, which have two target sites of miR390 with only the 3′-terminal target site usually sliced^11^. In contrast to the one-hit mode, the fragment upstream to the 3′ target site of miR390 is copied into dsRNA and processed by DCL4 from 3′ to 5′ into 21-nt phasiRNAs^11,12^. These phasiRNAs can function like miRNAs to regulate their target genes *in trans* (tasiRNAs) or *in cis* (casiRNAs).

### Major miRNA pathways

In general, plant miRNAs are classified into conserved miRNAs (present in angiosperms), less-conserved miRNAs (present in a lineage or group of plants), and species-specific miRNAs (present in a single species). A couple of miRNAs families are highly conserved while the majority are lineage-restricted or species-specific^3^. Here we collected 32 miRNA families to illustrate their conservation, including all the highly conserved ones in plants and a few families of horticultural importance.

**Fig. 2:**
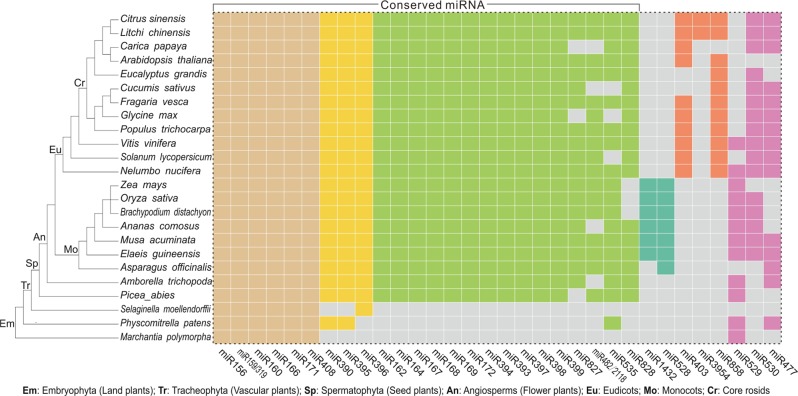
Conservation of miRNAs of horticultural importance. Dendrogram in the left panel denotes the rough evolutionary history of 24 species based on the APG (Angiosperm Phylogeny Group) IV system. Tile plot in the right panel presents the presence/absence of 32 representative miRNA families in the corresponding species. Gray-shaded tiles mean no presence or losing of the miRNA in the corresponding species while tiles filled with other colors denote the presence of the miRNA

It is estimated that the conserved miRNAs are composed of about 20 miRNA families that share distinct evolutionary routes^3^. Of them, nine appear to origin from land plants (embryophytes, Fig. 2) except with miR390 and miR395 families that are missing in liverwort (*Marchantia polymorpha*) and lycopod (*Selaginella moellendorffi*) and miR396 family that are missing in liverwort and moss (*Phycomitrella patens*), while the remaining 12 families derived from seed plants (spermatophytes, Fig. 2), except miR827 which was not found in gymnosperms (*Picea abies*). We also showed that three miRNA families including miR828, miR482/2118, and miR535, were widely present in seed plants but missed in a few lineages or species as evidenced by the absence of miR828 in grasses and miR482/2118 in papaya (*Carica papaya*) and cucumber (*Cucumis sativus*), respectively. Hence, miR828, miR482/2118, and miR535 should also be considered as the conserved miRNA families as well. The other eight miRNAs we listed seem to be lineage-restricted. For instance, miR1432 and miR528 are restricted in monocots, while miR403 is specific to eudicots (Fig. 2). Worthy of noting is that miR529 is missing in core rosids, but present in almost all ancient plants including liverwort and moss (Fig. 2).

The conserved miRNAs share the same target genes across a wide range of plants in general while less-conserved ones show species- or lineage-specific target genes (Table 1). Highly conserved miRNAs often play key roles in the regulation of plant growth and development. For example, the conserved miR156 family is involved in regulation of developmental timing or the vegetative-to-reproductive transition, by down-regulation of *SQUAMOSA PROMOTER BINDING PROTEIN-LIKE* (*SPL*) genes^13^. On contrary, lineage-specific miRNAs likely perform functions specific to certain plants or groups. One of the representative examples is miR528 that is monocot-specific and involved in defense against virus infection in rice^14^. Similarly, miR3954 that triggers phasiRNA biogenesis and potentially regulates flower programming via targeting *NAC* genes^15^, is only identified in Sapindale and its close relatives.

**Table 1:** Main target genes of miRNA families in Fig. 2

miRNA	Target	miRNA	Target
miR156	Squamosa-Promoter Binding Protein-Like gene (*SPL*)	miR393	Toll-Like Receptors (*TIR*)
miR159/319	*MYB*	miR397	Laccase (*LAC*)
miR160	Auxin Response Factor (*ARF*)	miR398	Copper Superoxide Dismutase (*CSD*)
miR165/166	Class III Homeodomain Leucine Zipper transcription factors (*Zip III*)	miR399	Phosphate Over accumulator (*PHO*)
miR170/171	Scarecrow-Like proteins (*SCL*)	miR828	MYB, Trans-acting siRNA gene 4 (*TAS4*)
miR408	Uclacyanin (*UCL*)	miR482/2118	Nucleotide Binding Site-Leucine-Rich Repeats (*NB-LRR*)
miR390	Trans-acting siRNA gene 3 (*TAS3*)	miR535	Squamosa-Promoter Binding Protein-Like Gene (*SPL*)
miR395	Sulfate transporter 2 (*SULTR2*)	miR827	Phosphate Transporter 5 (*PHT5*), Nitrogen Limitation Adaptation (*NLA*)
miR396	Growth-Regulating factor (*GRF*)	miR1432	ABRE-binding factor (*BZ-1*)
miR162	Dicer-Like Gene (*DCL1*)	miR528	Ascorbate Oxidase (*AO*)
miR164	*NAC*	miR403	Argonaute 2 (*AGO2*)
miR167	Auxin Response Factor (*ARF*)	miR3954	NAC
miR168	Argonaute 1 (*AGO1*)	miR858	MYB
miR169	Nuclear factor Y (*NF-Y*)	miR529	Squamosa-Promoter Binding Protein-Like Gene (*SPL*)
miR172	APETALA2 (*AP2*)	miR530	Argonaute 1 (*AGO1*)
miR394	F-box gene	miR477	GRAS domain-containing protein

### Major phasiRNA pathways

Besides miRNA, phasiRNA has been extensively investigated in plants in recent years and a few of phasiRNA pathways are highly conserved as well as conserved miRNAs. PhasiRNAs can be produced from both long noncoding and protein-coding genes (*PHAS* genes). The first several genes generating phasiRNAs (called *TAS* genes due to the *in trans* function of phasiRNAs) identified in Arabidopsis are all noncoding genes, including *TAS1–4* genes. *TAS1–2* and its trigger miRNA (miR173) are specific to Arabidopsis. The *TAS3* targeted by miR390 generates several tasiRNAs that target *AUXIN RESPONSIVE FACTOR* (*ARF*) genes^11,16^. This miR390-*TAS3*-*ARF* is prevalently conserved in almost all land plants^16^. The miR390*-TAS3-ARF* pathway, an indispensable regulatory component in auxin signaling, is of critical function in the regulation of plant growth and development, including leaf morphology, lateral root growth and developmental timing^16^. The *TAS4* gene likely absent in grasses is targeted by miR828 and produces tasiRNAs targeting *MYB* genes, which are associated with anthocyanin biosynthesis. With the growth of sRNA research in non-model plants, more and more protein-coding genes have been reported to generate profuse phasiRNAs as well, including those encoding NUCLEOTIDE BINDING LEUCINE-RICH REPEAT PROTEINS (NB-LRR), PENTATRICOPEPTIDE REPEAT PROTEINS (PPR), and MYB TRANSCRIPTION FACTORS (MYB), NAC TRANSCRIPTION FACTORS (NAC), Ca^2+^ ATPase, F-BOX CONTAINING PROTEIN (FBX)^10,17,18^. Many of these pathways are present in a wide range of plants. For example, miR482/2118 predominantly targets *NB-LRRs* (or noncoding transcripts in grasses) and triggers phasiRNA production in almost all seed plants^18,19^. And the phasiRNA production from *PPR* genes are universally observed in angiosperms^18,20^.

## Small RNA research in horticultural plants

**Fig. 3:**
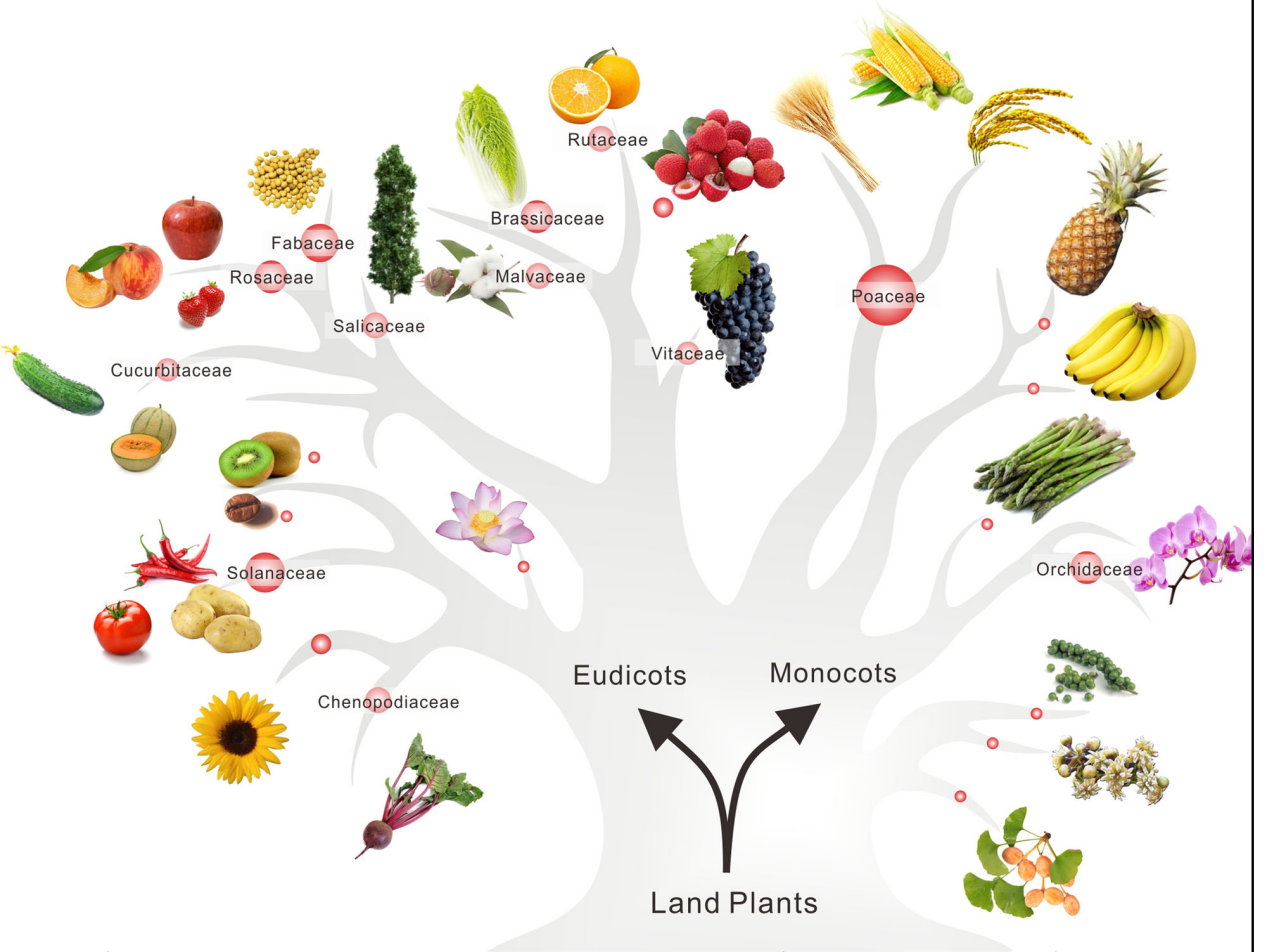
A draft tree representing main horticultural plants with sRNA studied. Pictures of representative species in plant families are posted on the tree drawn based on the APG (Angiosperm Phylogeny Group) IV system, with the size of red bubbles denoting the quantity of species. The bigger the bubble is, the more members of the plant family with sRNA datasets

To gain an overview of the scope of sRNA studies in plants, in particular the horticultural plants, we summarized the number of species in every plant families having sRNA deep-sequencing datasets deposited in the public repository NCBI-SRA (National Center for Biotechnology Information-Sequenced Read Archive, https://www.ncbi.nlm.nih.gov/sra). In general, most sRNA studies (with sRNA dataset deposited) focused on the economically important crops as illustrated in Fig. 3. Besides grasses (i.e., Poaceae), Solanaceae is the plant family with the largest number of species of sRNAs studied; it contains 12 species, including tomato^21^, pepper^22^, and potato^23^. The following is Fabaceae containing 11 species, which includes soybean^24^, Medicago^19^, and chicken pea^25^. Rosaceae, Brassicaceae, and Orchidaceae each has eight species in which sRNA population have been explored, while five species in each of Chenopodiaceae, Malvaceae, Rutaceae, and Salicaceae have sRNA datasets reported (Fig. 3).

For only a few plants of horticultural importance, their sRNA repertoire were relatively well profiled, including vegetables (tomato, potato, and cucumber^26^), fruits (grape^27^, citrus^15,28,29^, apple^30^, peach^31,32^, and strawberry^33^), and ornamental plants (petunia^34^ and orchid^35^). For fruit trees and ornamental plants, researchers focus their sRNA studies on processes related flowering time, fruit color pattern and fruit size^15,34,36–40^. In contrast, for vegetables, studies were mainly concerned about resistance to abiotic or biotic stresses that are directly associated with plant growth condition^41–44^. Here, we discuss several miRNA/phasiRNA-mediated pathways that are directly relevant to horticultural trait performance.

### Conserved miRNA or phasiRNA pathways and their regulation of horticultural traits

**Fig. 4:**
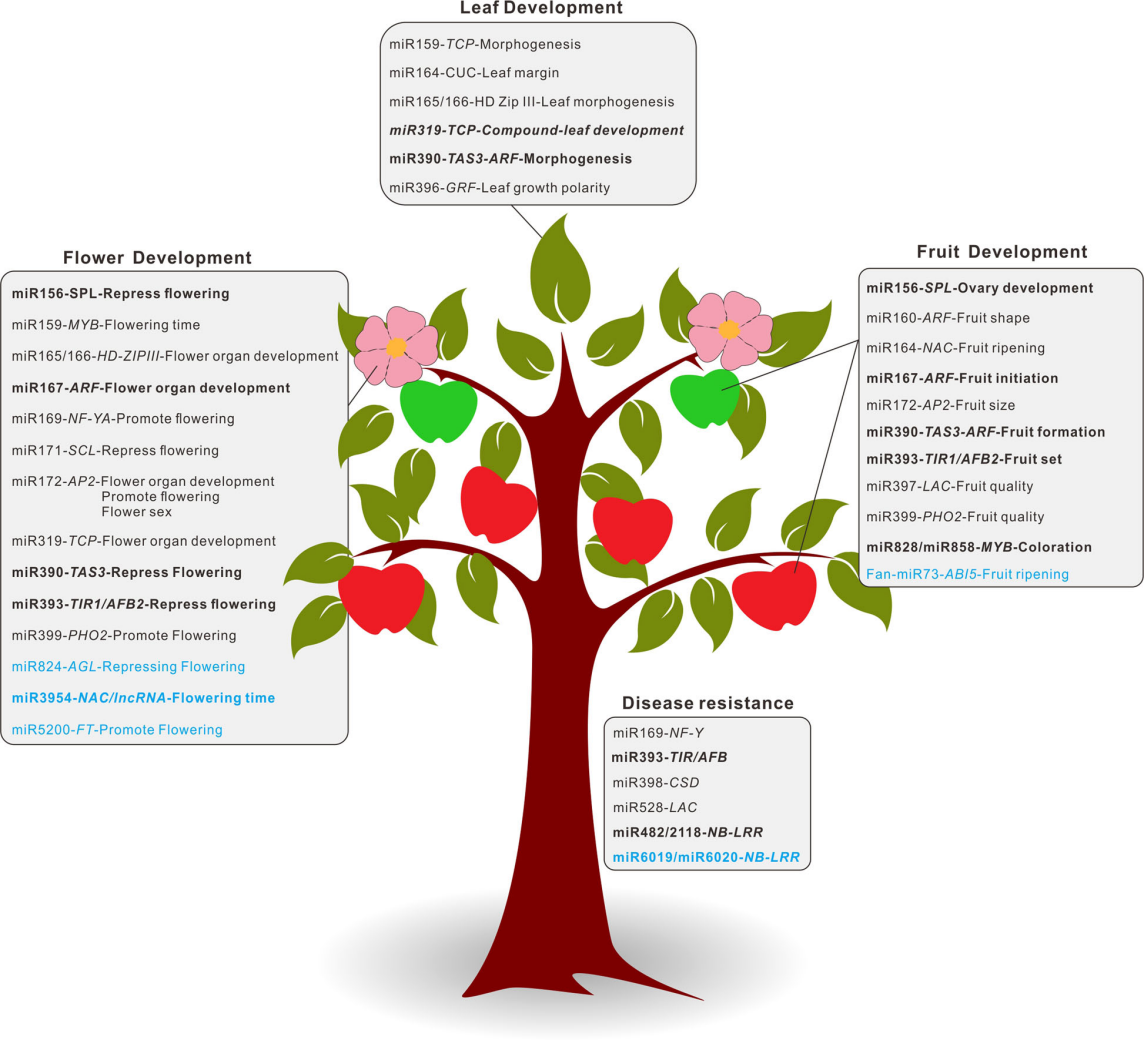
Main miRNA/phasiRNA pathways involved in the development of horticultural traits. Main miRNA/phasiRNA pathways are classified into four categories, including leaf development, flower development, fruit development, and disease resistance, which are illustrated in a cartoon apple tree. Pathways with phasiRNA integration are indicated in bold, and those lineage- or species-specific pathways are marked in cyan

In plants, conserved miRNAs usually play fundamental regulatory roles in plant growth and development. For horticultural plants, leaf (vegetables), flower (ornamental plants), and fruit (fruit trees) are usually the final products for harvest. Accordingly, as illustrated in Fig. 4, we classified the miRNA pathways in three major categories, leaf development, flower development, and fruit development, based on the main biological functions of miRNAs reported in the model plant Arabidopsis and a few other well-studied plants. In addition, we also added the category of disease resistance, as it is an indispensable part for healthy trait development of horticultural plants.

miR390, miR319, and miR396 are involved in the leaf development, including morphogenesis, growth polarity via regulating their target genes *TAS3-ARF*, *TCP*, and *GRF*, respectively. Many more miRNAs are associated with the development of flower and fruit^45–47^. For instance, miR165/166, miR172, and miR319 are essential for the flower organ development, and miR156, miR159, miR172, miR393, and many others are involved in the process of flowering time regulation^48–55^ (Fig. 4). In the fruit development, many conserved miRNAs, including miR160, miR167, miR172, miR390, miR393, miR828, miR858, participate in diverse part of fruit development, like fruit initiation, fruit size formation, fruit coloration, fruit ripening, etc.^39,56–62^ (Fig. 4). Regarding the disease resistance, the miR482/2118 superfamily are of vital roles via targeting a large number of *NB-LRR* resistance genes, which are a critical component of the effector-triggered immunity in plants^63,64^. Among these miRNA-involved regulatory pathways important for horticultural trait development, many are integrated with the generation of secondary phasiRNA (or tasiRNAs), which are believed to reinforce or broaden the downstream silencing effect of target genes, for instance, the miR390-*TAS3-ARF*, miR393-*TIR1/AFB2*, miR828-*MYB*, and miR482/2118-*NB-LRR*^16,19,30,65^.

A few miRNA pathways have a broad function, playing multiple roles in a few biological processes, which are different but interconnected. For instance, the miR390-*TAS3-ARF* is a critical regulatory circuit in the signaling pathway of a vital phytohormone auxin, thus the pathway is important for the development of all leaf, flower, and fruit; miR172 not only regulates the flower organ determination but also helps the fruit size formation. Therefore, miRNA-mediated regulations are sophisticated and interlinked; they can be associated with a wide range of processes important for the development of diverse traits. In the following discussion, we delineated a few major miRNA/phasiRNA-involved pathways, which have been studied relatively well in horticultural plants, to demonstrate their functional importance.

#### miRNAs-phase transition

**Fig. 5:**
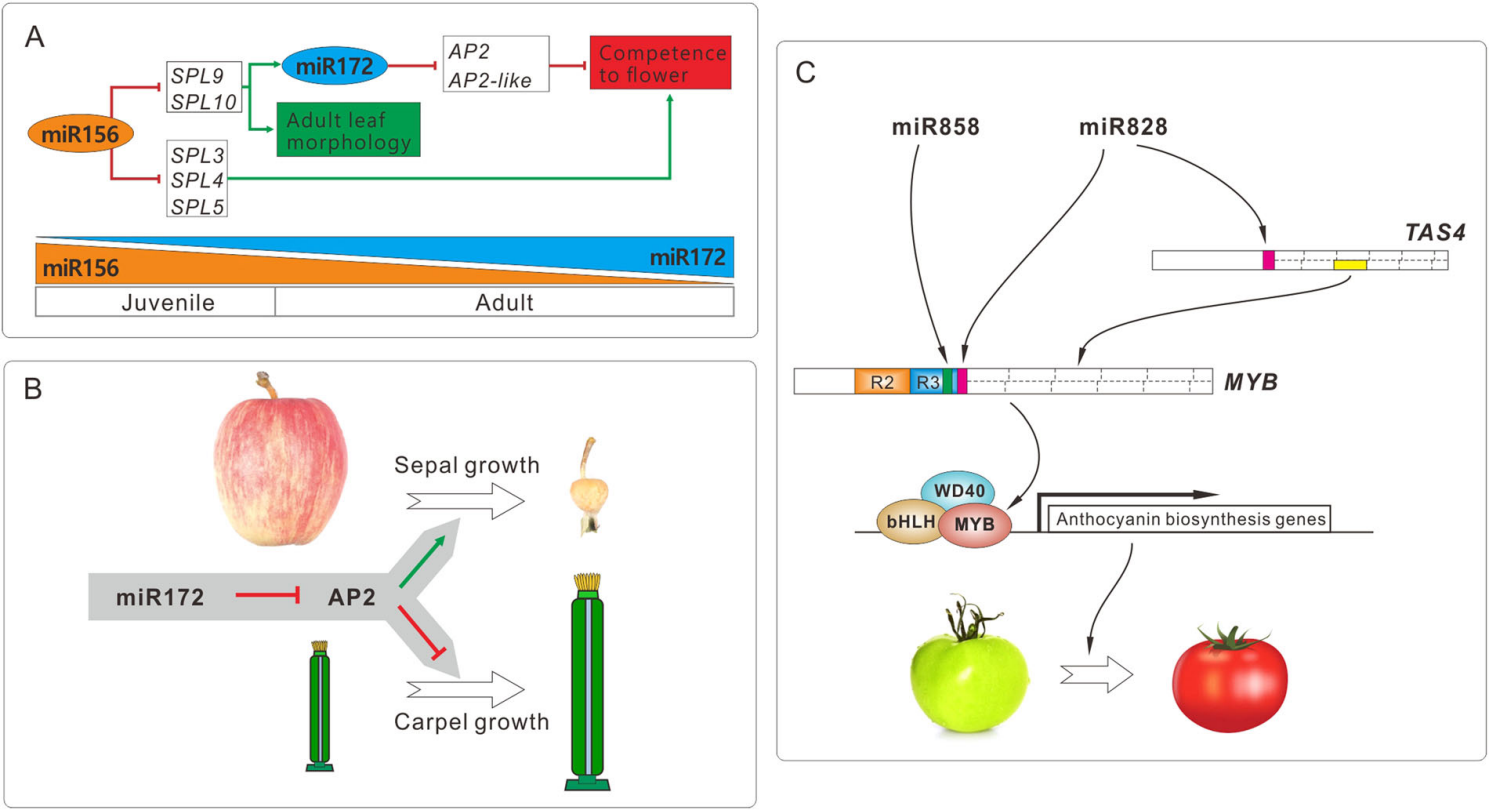
Representative miRNA/phasiRNA pathways functionally important in horticultural plants. **a** miR156 and miR172 cooperatively regulate the juvenile-to-adult phase transition in plants. MiR172, in collaboration with miR156, participates in the regulation of juvenile-to-adult phase transition in plants. These two miRNAs play antagonistic roles in flowering induction; high level of miR156 extends juvenile phase and delays flowering, while miR172 accumulation leads to early flowering^75^. **b** miR172 affects fruit size differently in apple and Arabidopsis depending on fruit type. Apple fruit is mostly derived from the hypanthium contributed most by sepal tissues which is positively regulated by *AP2* and over-accumulation of miR172 leads to the silencing of *AP2*, then leading to the dramatic reduction of fruit size and weight^39^. In contrast, the Arabidopsis fruit (silique) is derived from carpel tissues, in which *AP2* limits cell division and expansion; therefore miR172 overexpression (inhibiting the *AP2* expression) in Arabidopsis gives rise to bigger siliques^56^. **c** miR828 and miR858 function together to regulate the expression of *MYB* genes, which are involved in the pathway of anthocyanin biosynthesis, affecting fruit coloration^30^

MiR172 is conserved in plants and plays vital roles in plant development. It has been shown that miR172 repressed the expression level of *APETALA2* (*AP2*) or *AP2-like* genes by inhibiting translation or initiating degradation of the target mRNA^66–68^. MiR172 is important for the floral transition in many plants, including tomato, apple and so on^69–74^. MiR172, in collaboration with miR156, participates in the regulation of juvenile-to-adult phase transition in plants^75^. These two miRNAs play antagonistic roles in flowering induction; high level of miR156 extends juvenile phase and delays flowering, while miR172 accumulation leads to early flowering. In Arabidopsis, miR156 is highly abundant in young seedling and decreases with the phase transition, while miR172 has an opposite expression pattern, as miR156 represses the expression of the *MIR172b* gene via its targeted *SPL9* and *SPL10* genes (Fig. 5a). The other group of miR156-targeted *SPL* genes (*SPL3/4/5*) promotes the floral meristem identity transition to induce flowering^13^ (Fig. 5a). This complex regulatory cascade consisting of miR156, miR172, and their target genes is conserved in both annual and perennial plants, and miR156 and miR172 are closely correlated with the juvenile and adult phases of woody species^76,77^. Overexpression of miR156 in transgenic *Populus x canadensis* reduces the expression of miR156-targeted *SPL* genes and miR172, and dramatically prolongs the juvenile phase^76^. Long juvenile phase is a common issue for perennial fruit trees, in which it usually takes 4–8 years to finish the juvenile-to-adult transition. miR156-SPL and miR172-AP2 modules are important regulatory hubs in the control of this transition, therefore, they have been considered as potential elements to be engineered biotechnologically to shorten the juvenile phase of fruit trees^78^. On the other hand, once proceeded to the adult phase, perennial fruit trees normally flower one time a year. It always needs a balance between vegetative growth and reproduction to achieve stable flowering annually. Whether the miR156 and miR172 are coordinated similarly to regulate flowering during a yearly developmental cycle of fruit trees needs further investigation.

Besides miR156 and miR172, there are many other miRNAs involved in the control of plant flowering time as elucidated in the model plant Arabidopsis (as reviewed in ref. ^79^). As the majority of them have not been studied in horticultural plants, whether their function or regulatory pathways are similar or how conserved their function remains elusive.

#### miRNAs-fruit development

##### miR172-fruit size

In addition to its pivotal role in phase change, miR172 makes a great contribution in the process of fruit growth. Its target gene AP2 is a negative regulator of fruit ripening with evidence that knock-down of SlAP2a leads to orange color, split open and bumpy surface of fruits in tomato^80^. Moreover, recent studies found that the miR172-*AP2* pathway affects fruit size in different species depending on fruit type^57^ (Fig. 5a). In Arabidopsis, the fruit (silique) is derived from carpel tissues; its growth is negatively regulated by *AP2*. MiR172 inhibits the expression of *AP2* that limits cell division and expansion, therefore miR172 overexpression in Arabidopsis gives rise to bigger siliques^56^. In contrast, apple fruit is mostly derived from the hypanthium contributed most by sepal tissues which is positively regulated by *AP2* and over-accumulation of miR172 leads to the silencing of *AP2*, then leading to the dramatic reduction of fruit size and weight (Fig. 5b)^39^. In addition, as an ovary-derived flesh fruit, tomato is found to develop parthenocarpic seedless fruit with smaller fruit size when miR172 is overexpressed^57^. Therefore, fine-tuning the expression of miR172 might be a good strategy to produce the fruit of desirable size. But given the vital role of miR172 in flower development, it might be much complicated to modulate the miR172 expression only in fruit without the disturbance of flowering time and flower organ development.

##### miR828/miR858-fruit coloration

As mentioned above, miR828 targets *TAS4* to generate tasiRNAs regulating *MYB* genes (Fig. 5c). In addition, miR828 and miR858 work together to co-regulate a large number of *MYB* genes by directly targeting at the region encoding the conserved R3 domain of MYB proteins, and miR828 triggers the production of secondary phasiRNAs from targeted *MYB* genes to reinforce its silencing effects^30^ (Fig. 5c). Most of these regulated *MYBs* belong to the R2R3 class, a main component of the MYB-bHLH-WD40 protein complex, which is associated with diverse biological processes^81^, especially the biosynthesis of anthocyanin, one of the main pigments in plants. In Arabidopsis, overexpression of miR828 reduces anthocyanin accumulation by repressing genes encoding MYB transcription factors^82^. In tomato, miR858 plays a negative role in anthocyanin biosynthesis, and blockage of *MIR858* leads to increased anthocyanin accumulation by modulating the expression of *SlMYB7* and *SlMYB48*^61^, while another report demonstrates that miR858a represses the translation of *MYBL2* in Arabidopsis seedlings, as a positive regulator of anthocyanin biosynthesis^62^. However, in Rosaceae plants, the apple *MYB10* and its homologs in close species, which have central roles in fruit coloration^83^, do not have a good target site for miR828 and miR858, indicating that these two miRNAs likely play versatile or indirect roles in anthocyanin biosynthesis. As this pathway of miR828/miR858 targeting *MYB* genes is in Gymnosperm^18^, how this pathway is evolved with anthocyanin biogenesis thereafter is interesting to study. Conceivably, the miR8282/miR858 pathway is evolved with broader function with the expansion of *MYB* genes, as evidenced by their roles in fiber development^84^ and cyst nematode parasitism^85^.

##### miR397/miR399-fruit quality

Plant laccases, a large family of oxidases, are involved in lignin polymerization. Recently it was found that miR397 regulated fruit cell lignification in pear fruits by inhibiting expression of laccase gene^86^. A single nucleotide polymorphism (SNP) identified in the promoter of *PbrMIR397* gene is associated with low levels of fruit lignin^86^. This SNP may serve as a good genetic marker for the breeding selection of pear trees bearing fruits with low lignin content. In strawberry, fruits of high content of soluble solids are preferred by customer. Researchers found that the high content of soluble solids is positively correlated with high level of Pi content among different strawberry cultivars^87^. Phosphorus nutrition is a process under the regulation of miRNAs. The Pi-starvation responsive miR399 guides the cleavage of *PHO2* RNA, which encodes an E2 ubiquitin conjugase-related protein that negatively affects Pi content and remobilization^88^. Overexpression of miR399 can significantly improve fruit quality by increasing the Pi content and thereby the soluble solid content in strawberry fruit^89^. Higher soluble solids content is a common desirable trait for fruits. Whether this positive correlation of miR399 expression with soluble solid content is present in other types of fruits is worthy of an investigation.

As reported, miRNAs likely participate in every aspect of fruit development, from fruit set to fruit ripening, and from fruit size determination, fruit shape formation to fruit coloration. Although the function of conserved miRNAs is well-maintained among different plants, they are likely to have different effects on fruit quality, because “fruits” (eventual product for harvest) of many horticultural plants come from different organs, which are likely under a different regulation.

#### miRNAs-auxin signaling

**Fig. 6:**
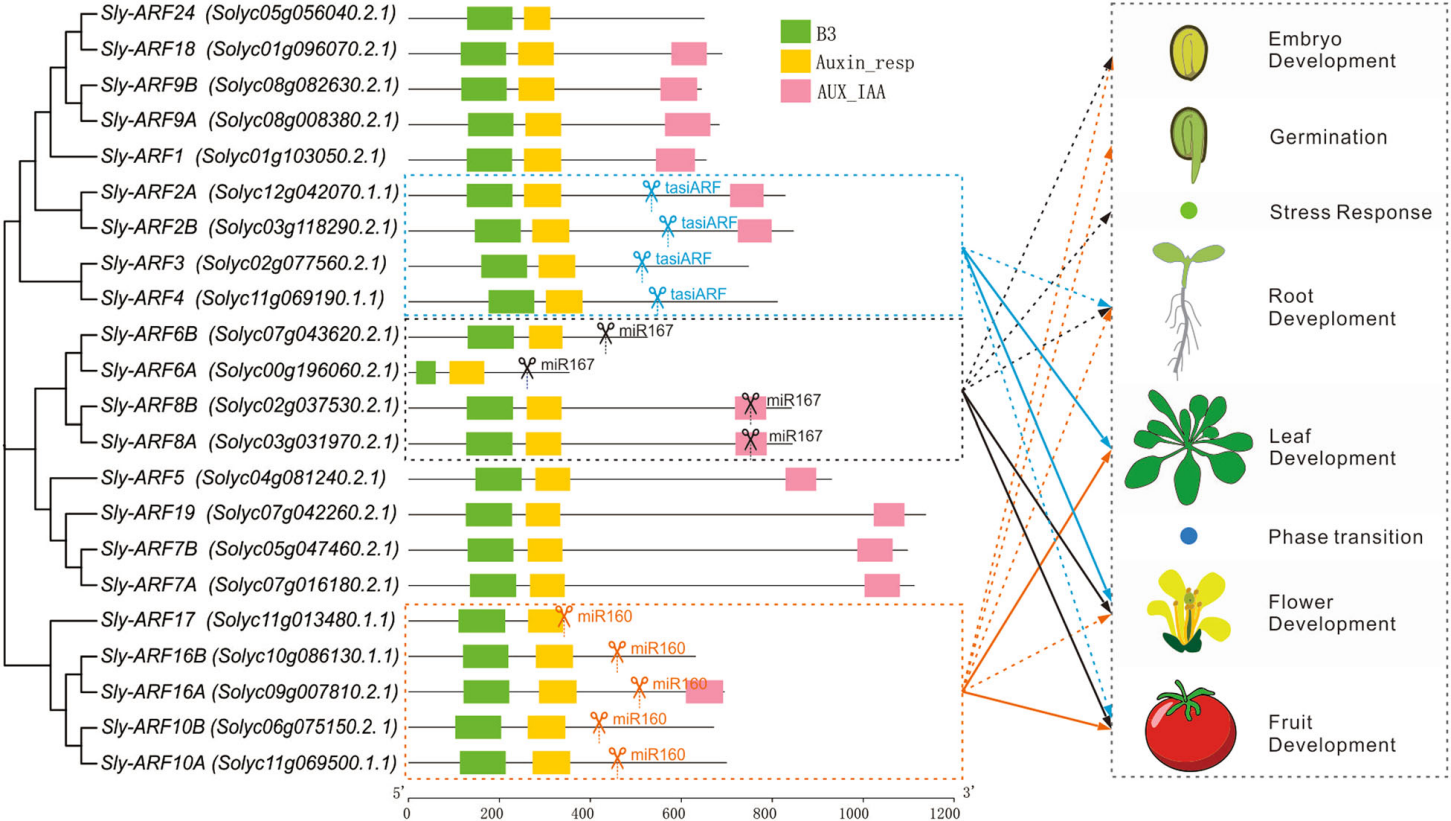
MiR160, miR167, and miR390 are involved in the regulation of *ARFs*. Left is the unrooted tree of tomato ARFs; the middle is the domain structure of ARFs in tomato with most of the ARFs consisting of three domains, B3 (green), Auxin_resp (yellow), and AUX/IAA (pink). Target sites of miR160, miR167, and tasiARF are marked respectively in their target genes; the right illustrates the biological functions of miR160/miR167/miR390-*ARF* pathways in plants, with thicker arrow denoting major functions of the corresponding pathway

Another important role of miRNAs and phasiRNAs is that they are involved in the signaling pathway of auxin, a key plant hormone regulating plant growth and development, through regulating the *AUXIN RESPONSIVE FACTORS (ARFs)*. ARFs, a class of transcription factors critical in auxin signaling, work together with Aux/IAAs in auxin-mediated growth and developmental processes by binding to the AUXIN RESPONSE ELEMENT (AuxRE) site in the promoter region of early auxin response genes. Many *ARFs* are regulated by miRNAs to trigger miRNA-mediated regulation of auxin responses in plant development. For example, there are 22 *ARFs* in tomato, falling into three clusters. There are a few members in each cluster regulated by miRNAs^90^ (Fig. 6). *ARF6/8* have been shown to be negatively regulated by miR167^91^, and *ARF10/16/17* are post-transcriptionally regulated by miR160^92–94^. Furthermore, as mentioned above, miR390 triggers the production of tasiRNA (tasiARF) from *TAS3* genes to target *ARF2/3/4*^6,95,96^. These three miRNA-mediated regulatory pathways are highly conserved in diverse plants^11,97^.

##### miR160-ARF10/16/17

MiR160 is involved in many biotic processes in plants, including flower identity specification, leaf development, fruit formation and etc.^7^. Upon sly-miR160 down-regulation using a short tandem target mimic (STTM160), its target genes ARF10/16/17 all up-regulate, and tomato fruits show elongated, pear-shaped morphology compared to control tomatoes due to the pre-anthesis shape alteration^98^. In addition, sly-miR160 down-regulation also alters the phenotype of vegetative lateral organs and inhibits the abscission of petal, anther, and fruit in tomato^98^. Ectopic expression of miR160-insensitive *SlARF10A* (*mSlARF10A*) results in narrow leaflet blades, sepals and petals, and abnormally shaped fruit; notably, transgenic fruits have a clear cone shape and are almost seedless with abnormal seeds that could not germinate^99^. Overexpression of the miR160-targeted *ARFs SlARF10A*, *SlARF10B*, or *SlARF17*, leads to reduced lamina and increased leaf complexity, and suppresses auxin response in tomato in young leaves^100^.

##### miR167-*ARF6/8*

MiR167 plays vital roles in the development of flower and fruit as well as root. MiR167 targeted *ARF6/8* regulate flower organ development^91^. Down-regulation of *ARF6* and *ARF8* by miR167 results in shorter petals, stamens, and leaves size, with the largest defects in floral development and female sterility^101^. Transgenic introduction of aberrant *ARF8* transcripts affects fruit initiation, leading to parthenocarpic fruit formation in both Arabidopsis and tomato^102,103^. MiR167 has also been implicated in plant immunity. MiR167 is down-regulated in response to fungal infection in Arabidopsis^104^ and during bacterial stress, miR167 alters the expression of genes of the host auxin signaling pathway, including *ARF6/8*^105^.

##### miR390-TAS3-ARF2/3/4

The miR390-*TAS3-ARF* pathway is mainly involved in the regulation of leaf and flower development, especially the leaf morphogenesis. The tasiRNA-mediated regulation of *ARF3* and *ARF4* is required for normal leaf morphogenesis; it stabilizes abaxial organ identity in *Arabidopsis thaliana*, tomato, and tobacco^45,95,106,107^. When tasiARFs fail to accumulate in tomato, misexpression of *ARF3* and/or *ARF4* leads to needle-like leaves in a species-specific manner, while reducing the activity of both *ARF3* and *ARF4* can rescue the wiry leaf lamina^108^. A collection of mutant plants of *AGO7*, a dispensable Argonaute partner of miR390 activity, including Arabidopsis, maize, tomato, Medicago, and monkey flower, show severe leaf and flower defects, for example, wiry leaves in tomato, and lobed and elongated leaves and abnormal flowers with defected organs in Medicago^45,109–111^. Overexpression of *SlARF2* in tomato results in pleiotropic morphological and developmental phenotypes, such as increased lateral root formation and flower organ senescence^112^.

In summary, these three miRNA-(tasiRNA)-*ARF* regulatory modules tend to have distinct main functions, i.e., the miR160-ARF10/16/17 module is important for the development of leaf and fruit, miR167-ARF6/8 essential for flower and fruit, and miR390-*TAS3-ARF2/3/4* for leaf and flower (Fig. 6). On the other hand, these three modules are intertwined with each other to have a common function. For instance, they all function in root development; *AtARF6/8* (targeted by miR167) and *AtARF17* (targeted by miR160) control adventitious rooting in Arabidopsis^113^. miR390 is regulating lateral root elongation by suppressing the expression of *ARF4* to allow the outgrowth of the emerging lateral root^114,115^. Auxin is a chemical widely used in almost all the aspects of the horticultural industry. miR160, miR167, and miR390 comprise three major regulatory hubs, adding more plasticity to the auxin signaling pathway. A good understanding of the roles of these hubs is of great significance for more effective and efficient application of auxin in the industry. In addition to auxin, miRNAs are involved in the metabolism or signaling of almost all other phytohormones, including ethylene, gibberellin, cytokinin, and abscisic acid (as reviewed in ref.^116^). But so far, studies regarding miRNA-involved phytohormone homeostasis in horticultural plants are very few.

#### miRNAs-disease resistance

miRNAs have also been demonstrated to play critical roles in many other aspects, especially stress responses^67,104,117–119^. miRNAs have been shown to be directly involved in regulation of disease resistance (R) genes^19,41,120^. Among them includes the miR482/2118 superfamily, which target a large number of *NB-LRR* genes^19,41^. In virus- or bacteria-infected tomato, the expression of miR482 is suppressed while some of its disease-resistant *NBS-LRR* target genes are up-regulated^120^. miR482/2118 is 22 nt long and has been demonstrated to trigger the production of 21-nt phasiRNAs from their targeted *NB-LRR* genes. Members of the miR482 family are down-regulated in cotton seedlings infected with a fungal pathogen *Verticillium dahliae*; they induce the expression of specific *NBS-LRR* genes in cotton, implying that miR482-mediated silencing of *NBS-LRR* genes is released in cotton upon fungal pathogen infection to activate disease defense^121^. miR482/2118-targeted *NB-LRR* genes comprise one of the largest gene families producing abundant phasiRNAs, a clear understanding of the role of these secondary phasiRNAs is still lacking. One possibility is that phasiRNAs is to maintain the low-level expression of *NB-LRR* genes in normal condition without pathogenic stresses^64^. miR528 and miR398 are also involved in resistance to virus or other biotic stresses^14,122^. They target a group of oxidases, including laccase, ascorbic acid oxidase, superoxide dismutase, which contribute to plant defense through the regulation of the level of reactive oxygen species.

### Lineage- or species-specific miRNA or phasiRNA pathways important in horticultural plants

Increasing studies have demonstrated that a few miRNAs restricted in certain plant lineages also play vital roles in various biological processes. Xia et al.^17^ characterized two clusters of miRNAs, which regulate a large number of *F-box* (*FBX*) genes from woodland strawberry (diploid), and one of these miRNAs is able to trigger subsequent phasiRNA production to reinforce the silencing of *FBX* genes. This miRNA-*FBX*-phasiRNA circuit targets an array of genes that are possibly involved in regulation of different biological events, including disease resistance and fruit development^17^. Another specific miRNA found in *Fragaria ananassa* (Octoploid) targets the *ABI5* (*ABA-INSENSITIVE 5*) gene, which encodes a critical transcription factor in the ABA signaling pathway; this regulation is likely involved in fruit ripening and responses to environmental stresses^123^. Recently, a study in citrus reported that 22-nt miR3954 targets a *NAC* transcript and two citrus-specific non-coding transcripts to trigger the biogenesis of phasiRNAs, which might be involved in the induction of early flowering in citrus^15^. This regulatory pathway has also been found in litchi^124^, a plant phylogenetically close to citrus, implicating that the miR3954-*NAC*/lncRNA-phasiRNA is likely a lineage-restricted pathway related to flowering induction. Lineage- or species-specific miRNAs represent a large class of sRNAs in plants. Although their function is not as fundamental or broad as conserved miRNAs, they are believed to be associated with the development of specific feature of certain lineage or species, like unique traits of horticultural plants.

### Other interesting studies on sRNAs in horticultural plants

In addition to miRNA and phasiRNAs, other sRNAs have also been reported to contribute to phenotypic diversity of horticultural plants. A study on persimmon (*Diospyros lotus*), a dioecy plant with heterogametic males (XY), identified a Y-specific sex-determinant candidate (OGI), which produces a sRNA targeting the autosomal MeGI gene, encoding a homeodomain transcription factor regulating anther fertility in a dosage-dependent fashion^125^. Another study investigating the formation of flower color pattern in snapdragon (*Antirrhinum majus*) found that an inverted duplication that generates sRNAs which repress a pigment biosynthesis gene, is the cause of population-wide differences in color patterns; the inverted duplication is under selection and is likely an intermediate on the pathway to miRNA evolution^126^. These sRNAs uncovered in these two exceptional studies do not belong to the miRNA or phasiRNA, or other well-known sRNA classes, demonstrating that the sRNA population and their function in plants are probably much more complicated than what we understand now.

## Concluding remarks

In the past one and a half decades, the rapid development of next-generation sequencing technologies stimulates an unprecedented sRNA research progress in plants in general, and horticultural crops in particular, because many of them (e.g., apple, peach, etc.) are not amenable to genetic analysis due to long juvenility and complexity genetics. It becomes apparent that horticultural crops share the conserved miRNA and phasiRNA pathways with other plants, and they also evolve their lineage- or species-specific miRNA/phasiRNA pathways, which have not been found even in other horticultural plants. Conserved miRNA/phasiRNAs often plays fundamental roles in processes important for healthy growth and normal development, for instance, flowering programming, fruit development, and disease resistance. Lineage- or species-specific miRNAs/phasiRNAs are biologically meaningful as well to researchers because they may confer or regulate the traits that other plants or crops lack or have not evolved. Hence, the elaboration of the relation between regulation of certain miRNA/phasiRNAs and expression of specific traits would provide invaluable information for practical breeding programs. Although a great progress has been made in sRNA research in horticultural crops, such progress is yet limited to a few species but have not been achieved in many other horticultural crops. Hence, continuous profiling and analysis of sRNAs, discovery of new miRNAs and unraveling of their regulatory pathways using computation-based approach are necessary. Ideally, these identified miRNAs need to be functionally validated in host plants through down-regulation using RNAi or CRISPR and up-regulation using ecotopical expression approaches. Unfortunately, performing such analyses in many horticultural plants remains challenging because of lack of an effective transformation system. In short, in the past decade we have just opened the door and have a glimpse of sRNAs in horticultural crops; further studies, including both exhaustive bioinformatics data mining and in-depth functional decoding, will be needed to uncover a more complete picture of them in the future.
